# Supplementary material for: Manipulation and control of droplets on surfaces in a homogeneous electric field
Source: Nat Commun. 2022 Jan 12;13:289. doi: 10.1038/s41467-021-27879-0 (PMC8755840; doi:10.1038/s41467-021-27879-0)
Supplement: Supplementary file 1 — Supplementary Information [file 41467_2021_27879_MOESM1_ESM.pdf]

Manipulation and Control of Droplets on Surfaces in a Homogeneous Electric Field
# – Supplementary Information –

Johannes Hartmann,[1,*] Maximilian T. Schür,[1,*] and Steffen Hardt[1,†]
[1] *Technische Universität Darmstadt, Fachbereich Maschinenbau,*
Fachgebiet Nano- und Mikrofluidik, Alarich-Weiss-Straße 10, D-64287 Darmstadt, Germany
(Dated: January 4, 2022)

CONTENTS

Supplementary Figures 2

Supplementary Tables 10

Supplementary Methods 11
- Experiments 11
  - Experimental setup 11
  - Long-range capillary interactions 11
  - Processing and analysis of experimental data 12
  - Validation of friction law 13
  - Volume of the mobile droplets 13
  - Verification of surface quality 13
- Numerical computations 13
  - Implicit approach to solve the Young-Laplace equation 13
  - Perfect dielectric vs. perfectly conducting droplets 15
  - Local distribution of electrostatic Maxwell stress for interacting droplets 15
  - Meshing and mesh convergence 16
  - Computation of the repulsion force based on the virtual work principle 16
  - Model of interacting dipoles 17

Supplementary References 18

* J.H. contributed equally to this work with M.T.S..
† To whom correspondence should be addressed: hardt@nmf.tu-darmstadt.de; https://www.nmf.tu-darmstadt.de

SUPPLEMENTARY FIGURES

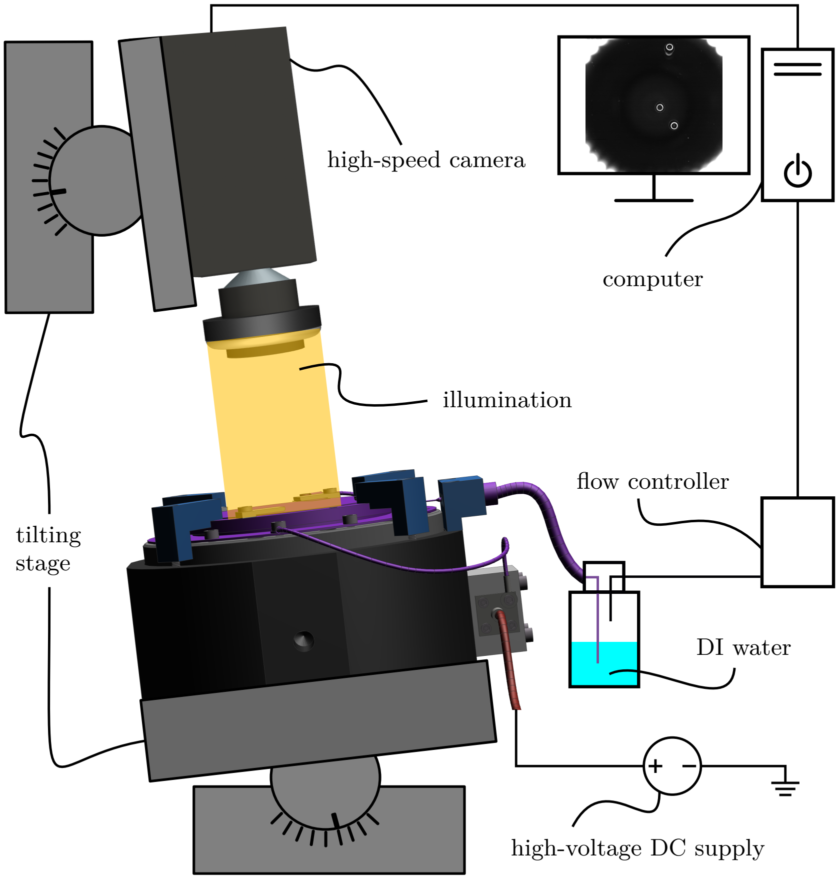

Supplementary Fig. 1. Schematic overview of the experimental setup.

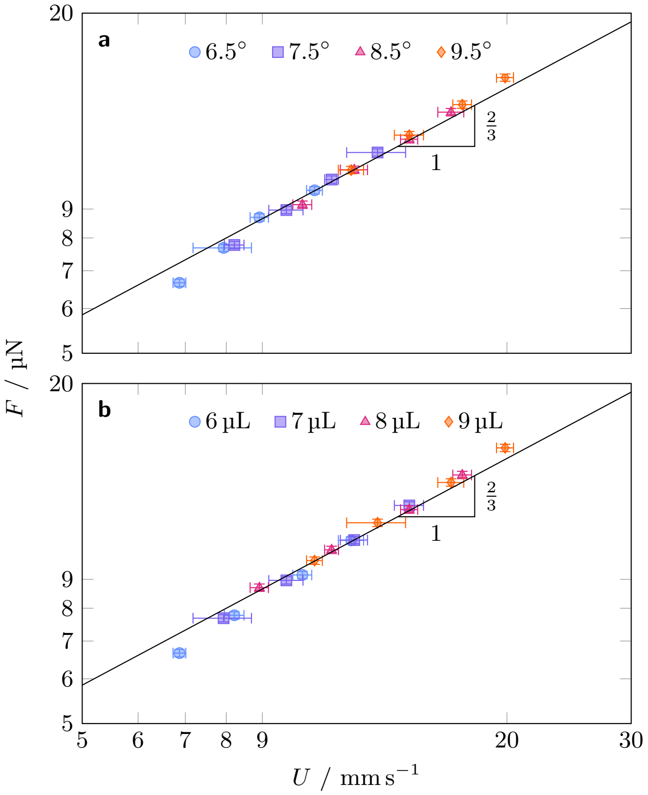

Supplementary Fig. 2. Log-log plots showing the friction force F as function of the velocity magnitude U. The data points are exactly the same in both plots. They only differ in the labeling of the symbols: In **a** the labels indicate the inclination angle $\alpha \in \{6^\circ, 7.5^\circ, 8.5^\circ, 9.5^\circ\}$, in **b** they indicate the droplet volume $V \in \{6.5\,\mu\mathrm{L}, 7\,\mu\mathrm{L}, 8\,\mu\mathrm{L}, 9\,\mu\mathrm{L}\}$. The straight solid line with slope $\frac{2}{3}$ represents the theoretical universal scaling law $F_\mathrm{r} \propto U^{\frac{2}{3}}$ proposed in [1]. The data points represent the mean of five single measurements. The corresponding standard error of the mean in F and U is visualized with error bars.

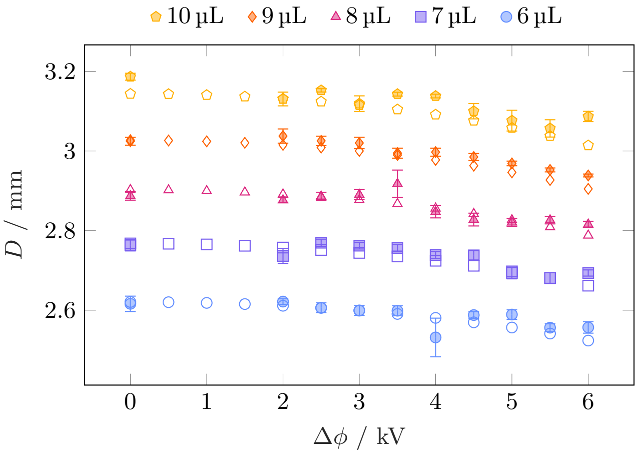

Supplementary Fig. 3. Lateral diameter D as a function of applied voltage $\Delta\phi$ for different droplet volumes ranging between 6 µL and 10 µL. Here, D is the diameter of the visible cross section of a droplet, as captured by the high-speed camera in top view. Experimental data are marked with filled symbols while the corresponding empty symbols represent numerical solutions of the Young-Laplace equation 8. Each experimental data point represents the average of ten single measurements. The error bars represent the standard error of the mean.

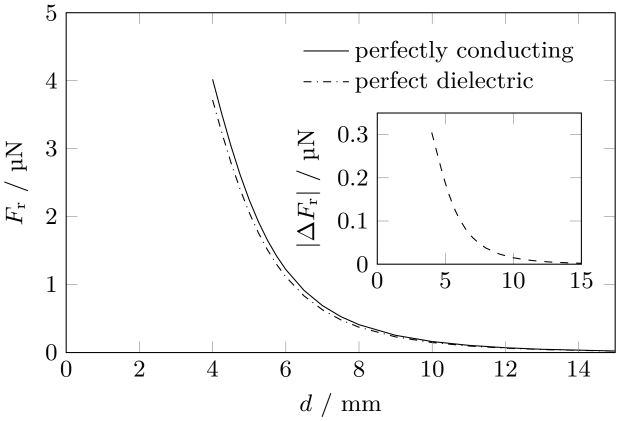

Supplementary Fig. 4. Comparison of the numerically computed repulsion forces between perfect dielectric and perfectly conducting water droplets with volumes $V_1 = 9.5\,\mu\text{L}$, $V_2 = 20\,\mu\text{L}$, and voltage difference $\Delta\phi = 4.5\,\text{kV}$. The inset shows the absolute difference between the computed forces.

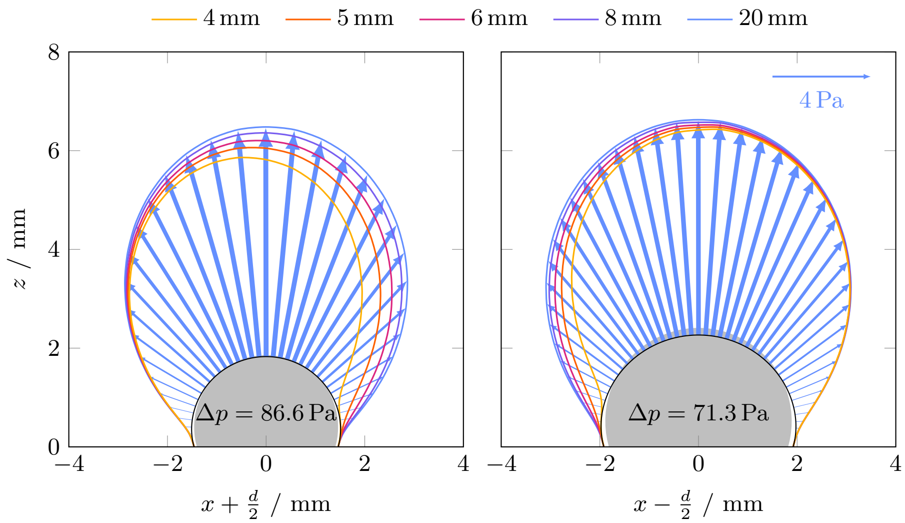

Supplementary Fig. 5. Numerically computed electrostatic Maxwell stress distributions in the symmetry xz-plane acting on the surface of two conducting droplets with volumes 9.5 µL (left) and 20 µL (right) for $d \in \{4\,\text{mm}, 5\,\text{mm}, 6\,\text{mm}, 8\,\text{mm}, 20\,\text{mm}\}$. The length and width of the arrows scales linearly with the magnitude of the electrostatic Maxwell stress. In the upper right corner a scale is provided. The maximum stress found at the apex of the smaller droplet is 8.74 Pa. The envelope of the stress distribution is color-coded for different d. The solid black droplet contour visualizes the axisymmetric equilibrium shape of the interacting, rigid droplets satisfying the Young-Laplace equation 8. For each droplet the corresponding equilibrium Laplace pressure Δp is specified. By contrast, the gray shaded areas illustrate the cross-sections of ideally spherical caps with the same volume. For $d \to \infty$ the stress distribution converges rapidly towards the axisymmetric distribution obtained for a single droplet deposited in the plate capacitor.

a

b

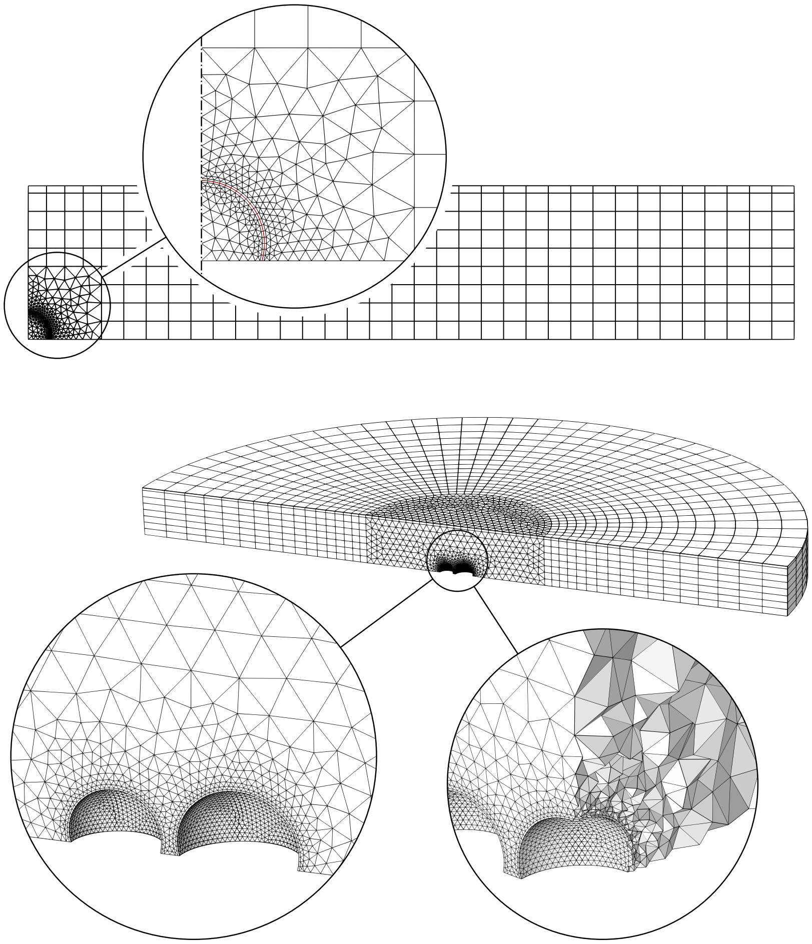

Supplementary Fig. 6. Tesselation of the computational domains for exemplary droplet sizes. **a** Axisymmetric computations of single-droplet equilibrium configurations in a parallel-plate capacitor: The interface of the droplet is highlighted in red while the dashed-dotted line represents the symmetry axis. The volume of the depicted droplet is 9.5 µL. **b** Three-dimensional computations of two rigid, axisymmetric, interacting droplets in a parallel-plate capacitor: The insets show enlargements of the droplets' vicinity. The boundary at the front is the symmetry boundary lying in the plane with $y = 0$. The droplets have volumes of 9.50 µL and 20 µL, and their separation distance d is 4 mm.

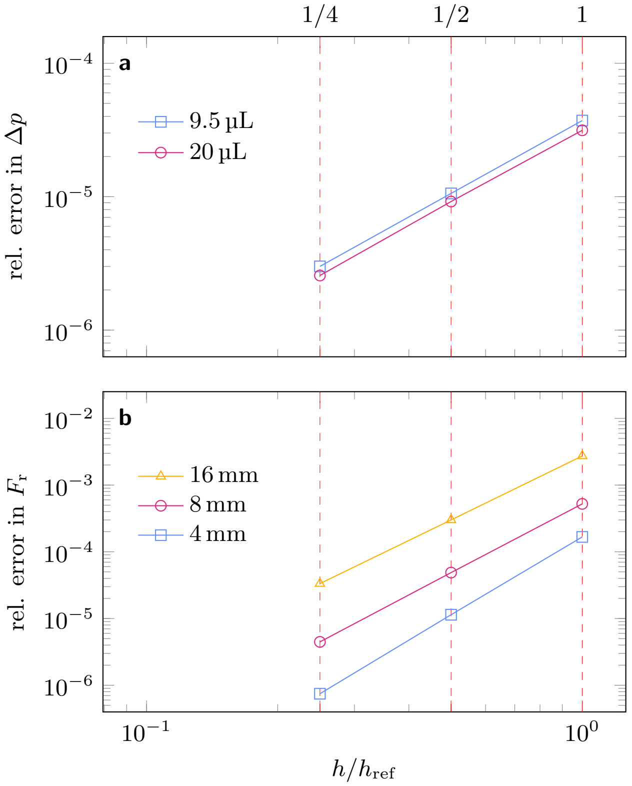

Supplementary Fig. 7. Mesh convergence of representative global quantities. **a** Axisymmetric computations of single-droplet equilibrium configurations in a parallel-plate capacitor: relative error in Laplace pressure $|\Delta p - \Delta p^*| / \Delta p^*$ as a function of the uniform, global mesh refinement level h/h_{ref}. Results for two different droplet volumes (9.5 µL and 20 µL) are shown. **b** Three-dimensional computations of two rigid, axisymmetric, interacting droplets in a parallel-plate capacitor: relative error in the repulsion force $|F_\mathrm{r} - F_\mathrm{r}^*| / F_\mathrm{r}^*$ based on Eq. 13 as function of the uniform, global mesh refinement level h/h_{ref}. Mesh convergence is shown for three fixed distances d between the droplets, coded with different symbols. The underlying parameters are $V_1 = 9.5$ µL, $V_2 = 20$ µL, and $\Delta\phi = 4.5$ kV. In both plots the solid lines connecting the data points are guides to the eye.

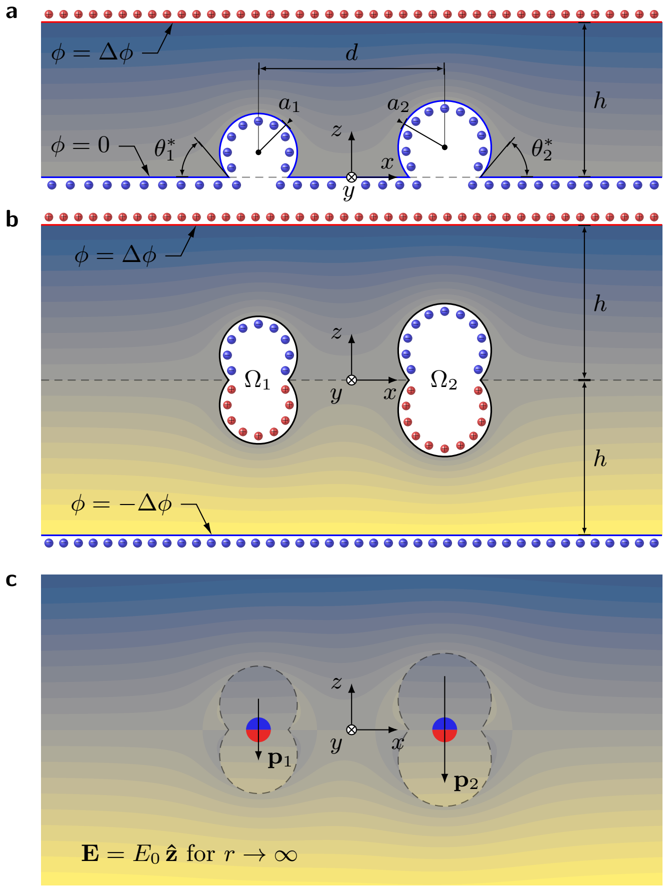

Supplementary Fig. 8. Visual representation of the model of interacting dipoles. All images show a cross-section of the three-dimensional domain along the symmetry plane at $y = 0$. **a** Original problem of two static sessile droplets placed in a parallel-plate capacitor. The droplets are supposed to be perfectly conducting. Between the electrodes at $z = 0$ and $z = h$ a constant voltage of $\Delta\phi > 0$ is applied. The free charges in the droplet and at the lower electrode right at the footprint cancel each other. Consequently, the footprint appears to be electrically neutral and the droplets are modeled as voids in the computational domain. Assuming perfectly spherical shapes the droplets are characterized by their radii a_1 and a_2 and their contact angles $\theta_1 = 180^\circ - \theta_1^*$ and $\theta_2 = 180^\circ - \theta_2^*$, respectively. The horizontal distance between the centers of the spherical caps is denoted d. Filled contours are used to visualize the numerically computed, scaled electrostatic potential $\phi/\Delta\phi$. The distribution of surface charges induced by the applied electric field is indicated by red and blue shaded symbols representing positive and negative charges, respectively. **b** Reformulated electrostatic problem involving two mirror-fused bodies, Ω_1 and Ω_2, placed in a parallel-plate capacitor with twice the plate spacing and applied voltage as in the original problem. This problem is equivalent to the original problem depicted in **a**. The electrostatic potential and the electric field are anti-symmetric with respect to the plane at $z = 0$. Consequently, the repulsion force between the mirror-fused spheres is twice the force between the spherical droplets in **a**. **c** Idealized problem of two induced interacting point dipoles with dipole moments $\mathbf{p}_1$ and $\mathbf{p}_2$ embedded in an unbounded medium with dielectric constant ε. The electrodes in problem **b** are substituted by a uniform electric field $E_0\hat{\mathbf{z}}$ far away from the droplets. The contours of the original mirror-fused bodies are indicated by the gray dashed lines. In the background the isocontours of the semi-analytical, piecewise-defined potential $\phi^*/\Delta\phi$ are plotted.

SUPPLEMENTARY TABLES

Supplementary Table I. Contact angles of 6µL DI water droplets on the LIS used in the experiments.

wafer no.	reconditioning cycle	equilibrium contact angle / °	advancing contact angle / °	receding contact angle / °
1	2	106.11±0.85	108.00±1.15	104.36±0.89
1	61	107.42±0.28	106.94±0.54	102.44±0.69
2	2	107.67±0.38	109.68±0.56	105.11±0.46

SUPPLEMENTARY METHODS

Experiments

Experimental setup

Figure 1 shows a schematic of the experimental setup. The part colored in purple represents the capacitor with electric connections and the mobile liquid feed line. This part is explained in detail in the main text.

The nozzles (Vieweg) protruding into the plate capacitor were made from polypropylene to suppress electric discharges between the nozzle and the capacitor plates. The droplets were generated by a digitally controllable micropump (Elveflow OB1 pressure controller Mk2) that allows controlling the pumping pressure. By selecting the nozzle size and the applied pressure, the droplet size and droplet frequency could be roughly predetermined. Other factors that could influence the droplet volume, such as the electric field and the angle of inclination of the LIS, prevented exact dosing of the droplet volume. Therefore, the calibration process described in section *Volume of the mobile droplets* was necessary to determine the exact droplet volumes in the repulsion force measurements. The position of the nozzle was chosen in such a way that coalescence could be expected if the moving droplet approaches the immobilized one. Nevertheless, owing to inaccuracies coalescence between the two droplets was not always achieved. Moving droplets that were shed in a way that they were able to pass the immobilized droplet within a center-to-center distance of maximal 1.5 times of the immobilized droplet diameter, even without the electric field switched on, were included in the data evaluation.

In all experiments, we used deionized (DI) water from a Millipore device (Milli-Q, $18.2\,\mathrm{M\Omega\,cm}$) as the droplet liquid. For applying the electric field at the capacitor we used a high-voltage source (Heinzinger PNC 6000 - 100). The high-voltage gate was connected to the glass wafer coated with indium tin oxide (ITO). The LIS was connected to the ground gate via the clamping ring and the silicon wafer base which is a milled aluminum block.

The aluminum block is electrically insulated from the optical table by a thick polyoxymethylene (POM) block. To be able to tune the in-plane gravitational acceleration, the POM block was mounted on a tilting stage with a range of $60°$ around one horizontal axis (accuracy of $10'$). Additionally, the tilting stage could be rotated by $360°$ degrees around its vertical axis (accuracy of $10'$). The second horizontal axis was aligned perpendicular to the gravitational field. The tilting stage on which the camera was mounted was a self-build freely movable and adjustable support. That way it was possible to adjust the camera's optical axis parallel to the normal vector of the LIS. A cross-line laser mounted on the camera sled was used to ensure this alignment. Subsequently, the alignment was verified with a calibrated try square.

The imaging system consists of a Photron high-speed camera (Photron Motion Pro Y4), a 32 mm extension tube and a lens (Nikon AF Nikkor 50 mm $1:1.4D$) to ensure a large field of view and a sufficient optical resolution at the same time. The illumination was provided by a coaxial LED ring light (Walimax Pro Makro).

Long-range capillary interactions

The estimation of the lateral extension of the wetting ridge is necessary to exclude that capillary forces between two droplets play a role in the experiments. These capillary forces become relevant if the wetting ridges of two droplets start to overlap, as explained by Kajiya et al. [2]. If the smallest measured distance between the droplets is bigger than the sum of the maximal lateral extensions of the wetting ridges of these droplets, capillary forces can be ignored in our considerations. In the following r_{ext} is a measure for the lateral extension of the wetting ridge.

We adopt the definition of r_{ext} from Kreder et al. [3], where r_{ext} is the radius of a circle fitted to the lubricant/air interface in the vicinity of the wetting ridge. Furthermore, let h_{f} denote the height of the lubricant layer behind a moving droplet. To estimate r_{ext} we refer to Kreder et al., who showed that the ratio $h_{\mathrm{f}}/r_{\mathrm{ext}}$ scales as a specific power Ca^x of the capillary number of the droplet, with x depending on the magnitude of Ca. Here, the capillary number Ca is calculated with the interfacial tension γ_{ld} between the lubricant and the droplet liquid. Kreder et al. provide an extensive data set for the range $10^{-4} < Ca < 1$ and find that $h_{\mathrm{f}}/r_{\mathrm{ext}} \propto Ca^{2/3}$ for $10^{-4} < Ca < 10^{-2}$ and $h_{\mathrm{f}}/r_{\mathrm{ext}} \propto Ca^{1/4}$ for $10^{-1} < Ca < 1$. This scaling was derived from the classical Landau-Levich-Derjaguin model and only holds for $r_{\mathrm{ext}} \ll R$, where R is the characteristic macroscopic length scale of the droplet. In our experiments, with a maximal droplet velocity of $20\,\mathrm{mm\,s^{-1}}$ and a silicone oil dynamic viscosity of $4.6\,\mathrm{mPa\,s^{-1}}$ (kinematic viscosity of 5 cSt), we obtain a maximal value for Ca of $2.3 \cdot 10^{-3}$.

To get an approximation for the initial thickness of the lubricant film before droplet deposition we refer to Emslie et al. [4], who calculated the flow of a liquid film on a rotating disk. Based on their findings the film thickness h^* of

the spin-coated lubricant in our experiments can be estimated by

$$h^* = \frac{h_0}{\sqrt{1 + \frac{4\rho\omega^2}{3\eta} h_0^2 t}} \,, \tag{1}$$

where ρ is the density of the silicone oil, η its dynamic viscosity, ω the angular frequency of the spin-coating process and t the processing time. The quantity h_0 is the initial silicone-oil layer thickness before the spin coating process, which we estimate to be 63.6 µm as we apply 500 µL silicone oil on a silicon wafer with a diameter of 100 mm. Using Eq. 1 we get $h^* = 2.4\,\mu\text{m}$ for the lubricant height after the spin coating process.

As we want to estimate the maximal lateral extension of the wetting ridge in the repulsion force measurements, we take h^* as an upper bound for h_f. This assumption is justified as we observe thin-film interference behind a moving droplet, which indicates that the actual h_f is smaller than 1 µm and thus smaller than h^*. Furthermore, Kreder et al. showed that the ratio $h_\mathrm{f}/r_\mathrm{ext}$ does not depend on the lubricant layer thickness in front of a moving droplet. Following these arguments and referring to the data of Kreder et al. we obtain a maximal lateral extension of the wetting ridge of $r_\mathrm{ext} = 96\,\mu\text{m}$ for a moving droplet corresponding to our experiments. For a sessile droplet, Kreder et al. measure a wetting ridge of 50 µm after applying the droplet to the LIS and show that the size of the wetting ridge rises linearly to 150 µm in 1800 s, under similar circumstances as in our experiments. In the present experiments, the immobilized droplet sits for the maximal time of only 120 s on the LIS before the experiment is finished. Therefore we argue that the wetting ridge of our immobilized droplet is smaller than 75 µm. Accordingly, if the droplets' surfaces are less than 171 µm apart from each other, their wetting ridges start to overlap. However, in our experiments, the droplets never got that close. More specifically, the gap width was measured to be always larger than 200 µm. Thus it is justified to neglect the attractive capillary forces between the droplets both in the analysis of the experiments as well as in the numerical computations.

Processing and analysis of experimental data

This section is concerned with the data obtained from the droplet repulsion experiments. Once recorded, the images need to be processed to obtain the desired volumes (masses) of the droplets, their centers of mass in the xy-plane, their trajectories, and their velocities and accelerations. We will describe one single experiment which is exemplary for the rest.

A single droplet interaction event corresponds to 250 to 300 frames recorded with the high-speed camera, depending on the velocity of the mobile droplet. All of the frames are background-corrected by subtracting a frame taken at the beginning of the same experiment without a moving droplet, to eliminate disturbing light reflections. The pixel width of the moving droplet is then measured by eye and subsequently translated into a volume, using the experimentally determined relation between projected droplet diameter and volume (see below). To detect the droplet's center in a single frame, we used the MATLAB® [5] Canny edge detector and the functions `imfill` and `centroid`. The output of these functions gives the position of the center of the first light reflection on the droplet surface $\mathbf{RC} = (RC_x, RC_y)$, which is visible in the Supplementary Movies 1 and 2 as the bright broad ring, located inside the striped ring marking the droplet's circumference. The circumference could not be used for tracking the center of the droplet as it could not be tracked reliably by the above mentioned MATLAB® functions. Because of this problem, a function had to be found that describes the relationship of the center of the droplet to the center of the automatically traceable reflection. We found this function by comparing the droplet's center $\mathbf{CC}$ as determined from the circumference and the reflection center $\mathbf{RC}$. For that purpose $\mathbf{CC} = (CC_x, CC_y)$ was tracked by eye. $\mathbf{CC}$ and $\mathbf{RC}$ were related by an appropriate fit function of the form $\mathbf{CC} = f(\mathbf{RC}, R_\mathrm{r}) + \mathbf{RC}$, where R_r represents the radius of the reflection ring with the corresponding center point $\mathbf{RC}$. The function

$$f(\mathbf{RC}, R_\mathrm{r}) = \frac{R_\mathrm{r}}{2} \begin{pmatrix} \sin((508\,\mathrm{px} - RC_x)/400\,\mathrm{px}) \\ \sin((508\,\mathrm{px} - RC_y)/400\,\mathrm{px}) \end{pmatrix} \tag{2}$$

gives an output matching the eye-tracked $\mathbf{CC}$ with a deviation of ± 1 pixels for the parameter space of the experiments. All arguments of the fit function are entered as pixel values. The fit function was determined based on 20 representative experiments. Subsequently, this function was used to automatically track the droplet's center in all experiments.

As 300 frames per second were recorded, the velocity of a moving droplet is of the order of $10\,\mathrm{mm\,s^{-1}}$ and the spatial resolution is 40.8 µm/px in the focal plane, the center of the droplet translates by about one pixel per frame, which means that between successive frames the imaging resolution is reached. This can lead to large unphysical fluctuations in the recorded velocities, given that the velocity is calculated based on two successive frames. This problem can be solved by fitting cubic smoothing splines to the time series of the center position of a moving droplet.

The velocity and the acceleration of the droplet can then be calculated by differentiation of the splines. Once the droplet velocity as a function of time has been determined, the forces acting on the droplet can be calculated using an in-house MATLAB® code, as described in the main text.

Validation of friction law

For detailed results of the measurements that confirm the universal friction law on LIS for the conditions of our experiment, the reader is referred to Fig. 2 that shows the friction force as a function of droplet velocity. In addition to that, the universal friction law was already confirmed on similar LIS for glycerol/water mixtures [6].

Volume of the mobile droplets

Our approach of determining the volume of the mobile droplets in the repulsion force measurements is briefly explained in the main text. Calibration measurements were conducted to determine the relation between the droplet volume, the projected diameter imaged in top view and the applied voltage. The droplet volume was varied between 6 µL and 30 µL with an increment of 1 µL. We recorded the deformation of the droplets for zero applied electric field and voltages ranging from 2 kV to 6 kV with an increment of 0.5 kV. The diameters of the recorded projection of the droplets, in units of pixels, were measured with the MATLAB® [5] function `imdistline`. Using the geometric camera calibration, the volume of a droplet could then be clearly assigned to the diameter of its projection. Smoothing splines were fitted to the discrete calibration data points to obtain a continuous and smooth relation between the lateral diameter, the droplet volume, and the applied voltage $\Delta\phi$. This improved the resolution with which we could distinguish between different droplet volumes in the repulsion force measurements. Results of the calibration measurements can be seen in Fig. 3. The calibration data used for the determination of droplet volumes in the repulsion experiments were extracted from the corresponding spline fits.

Verification of surface quality

To ensure the reproducibility of the experiments, the LIS needs to be refreshed before each experiment to minimize the influence of preceding droplets and to provide a homogeneous lubricant thickness. Although the preparation of the LIS is comparatively easy, it is still a limiting factor since it had to be repeated several hundred times to perform all necessary experiments. To show that repeated cleaning and relubrication of the PDMS-coated wafers do not alter the surface properties, we measured the equilibrium, advancing and receding contact angles on different wafers after different numbers of reconditioning cycles. The results of these contact angle measurements using a Krüss Drop Shape Analyzer DSA100 are shown in Table I. Each provided value represents the average of 50 single measurements. All measurements in the same row were conducted on the same individual wafer between two reconditioning steps, while different spots on this LIS were chosen for every single measurement. As can be clearly seen, the measured contact angles differ marginally between different wafers as well as different reconditioning cycles. This observation justifies our approach of using a total of three individual tempered wafers that are cleaned and relubricated after every single experiment.

Numerical computations

Implicit approach to solve the Young-Laplace equation

Coupling the Young-Laplace equation with electrostatics is not straightforward. It involves mapping of the Young-Laplace equation defined in the two-dimensional parameter space of the liquid surface onto the three-dimensional Euclidian space, where the equations of electrostatics are solved, and vice versa. To avoid these difficulties we instead adopt the dynamic approach presented in [6]. Here, the fluid dynamics inside a droplet is coupled with electrostatics by a dynamic stress balance imposed at the droplet/air interface. The latter is modeled explicitly as a sharp interface (discontinuity) between the droplet liquid and the ambient fluid. Within the arbitrary Lagrangian-Eulerian framework, which we rely on in the present work, a moving mesh is used to track the interface in space and time [7]. Starting with droplets of arbitrary shapes with specific volumes, the corresponding system of coupled equations is integrated in time until the interface has reached its static equilibrium. In the asymptotic limit the dynamic equations reduce to the Young-Laplace equation we aim to solve. In other words, we translate the steady problem of finding the static

equilibrium into an unsteady problem of finding the droplet shape in the asymptotic limit $t \to \infty$. In the following we will briefly discuss the governing equations of this problem.

Hereinafter, the subscripts "a" and "d" refer to the ambient and droplet phase, respectively. However, they might be dropped for ease of notation if the assignment is clear from the context. For a schematic of the computational domain we refer to Fig. 6 of the main text. First of all, we note that any non-linear phenomena in the fluid dynamics can be neglected without loss of generality, since we are only interested in the long-time limit where the velocity vanishes, i.e. $\mathbf{u} \to 0$. Thus for our purpose, the Stokes equations for the solenoidal velocity field $\mathbf{u}$ and the pressure p are perfectly sufficient:

$$\boldsymbol{\nabla} \cdot \mathbf{u} = 0 \ , \quad \rho^* \, \partial_t \mathbf{u} = -\boldsymbol{\nabla} p + \mu^* \boldsymbol{\nabla}^2 \mathbf{u} - \Delta\rho g \hat{\boldsymbol{z}} \ . \tag{3}$$

Here and below, all parameters equipped with an asterisk have no physical meaning in the long-time limit, as we shall discuss later. The last term on the RHS represents the gravitational volume force and takes into account the density difference $\Delta\rho = \rho_\mathrm{d} - \rho_\mathrm{a}$ between the droplet and ambient fluid. In the present problem the density of the ambient air is negligibly small compared to that of the droplet, such that $\Delta\rho$ can be replaced by $\rho_\mathrm{d} = 997\,\mathrm{kg\,m^{-3}}$. Right at the idealized three-phase contact line (TCL) the shear stress owing to slippage and the viscous flow within the droplet is balanced by the uncompensated Young stress, represented by the Generalized Navier Boundary Condition (GNBC)

$$\beta^* \, \mathbf{u} \cdot \hat{\boldsymbol{\tau}}_i + (\mathbf{T}^\mu \cdot \hat{\mathbf{n}}) \cdot \hat{\boldsymbol{\tau}}_i = -\tilde{\gamma} \left(\cos\tilde{\theta} - \cos\tilde{\theta}_\mathrm{s} \right) \hat{\mathbf{t}} \cdot \hat{\boldsymbol{\tau}}_i \ , \tag{4}$$

where $\tilde{\gamma}$ and $\mathbf{T}^\mu = \mu^* \left(\nabla\mathbf{u} + (\nabla\mathbf{u})^T \right)$ denote the effective interfacial tension assigned to the droplet/air interface and the viscous stress tensor, respectively [7]. The vector $\hat{\mathbf{n}}$ is the outward-pointing normal vector at the droplet surface, while the two vectors $\hat{\boldsymbol{\tau}}_i$ with $i = 1, 2$ are tangential to the interfaces. The vector $\mathbf{t}$ is perpendicular to the TCL and lies in the horizontal xy-plane. This boundary condition implies that in general the actual, dynamic apparent contact angle $\tilde{\theta}$ differs from its prescribed static counterpart $\tilde{\theta}_\mathrm{s}$. At the footprint of the droplet $\partial\Omega_{\mathrm{d/le}}$ the tangential velocity components satisfy a common Navier (slip) boundary condition (NBC)

$$\beta^* \, \mathbf{u} \cdot \hat{\boldsymbol{\tau}}_i + (\mathbf{T}^\mu \cdot \hat{\mathbf{n}}) \cdot \hat{\boldsymbol{\tau}}_i = 0 \ , \tag{5}$$

while the normal velocity component vanishes due to the kinematic boundary condition $\mathbf{u} \cdot \hat{\mathbf{n}} = 0$. In conjunction with the GNBC this condition ensures that the TCL can slip over the solid substrate. Fluid dynamics and electrostatics are mutually coupled by a dynamic stress balance at the deformable droplet/air interface $\partial\Omega_{\mathrm{d/a}}$

$$\left[(p - p_\mathrm{a}) \ \mathbf{I} - \mathbf{T}^\mu \right] \cdot \hat{\mathbf{n}} + \mathbf{f}^\phi = \tilde{\gamma} \ (\boldsymbol{\nabla} \cdot \hat{\mathbf{n}}) \, \hat{\mathbf{n}} \ , \tag{6}$$

where the electrostatic surface force density $\mathbf{f}^\phi = [\![\mathbf{T}^\phi]\!] \cdot \hat{\mathbf{n}}$ acting on the droplet/air interface with curvature $\kappa = \boldsymbol{\nabla} \cdot \hat{\mathbf{n}}$ is expressed in terms of the discontinuity of the electrostatic Maxwell stress tensor $[\![\mathbf{T}^\phi]\!]$ across the interface with

$$\mathbf{T}^\phi \equiv \varepsilon \, \mathbf{E} \otimes \mathbf{E} - \frac{1}{2} \varepsilon \, (\mathbf{E} \cdot \mathbf{E}) \ \boldsymbol{\delta} \ . \tag{7}$$

In the equation above $\mathbf{E}$ is the electric field, $\varepsilon = \varepsilon_0 \varepsilon_r$ represents the absolute permittivity and $\boldsymbol{\delta}$ denotes the identity matrix. With $\mathbf{T}^\phi_\mathrm{d}$ and $\mathbf{T}^\phi_\mathrm{a}$ denoting the local electrostatic Maxwell stress tensor approaching the interface from the side of the droplet and the ambient phase, respectively, the discontinuity can be expressed as $[\![\mathbf{T}^\phi]\!] = \mathbf{T}^\phi_\mathrm{a} - \mathbf{T}^\phi_\mathrm{d}$. Assuming perfectly conducting droplets and $\varepsilon = \varepsilon_0$ in the ambient phase the electrostatic surface force density $\mathbf{f}^\phi$ simplifies to $\frac{1}{2} \varepsilon_0 \, (\mathbf{E} \cdot \mathbf{E}) \, \hat{\mathbf{n}}$, as can easily be shown by exploiting the fact that the electric field inside the droplet vanishes ($\mathbf{E} = 0$), while it is purely normal within the ambient phase right at the interface, i.e. $\mathbf{E} \cdot \hat{\boldsymbol{\tau}}_i = 0$ with $i = 1, 2$.

In general the velocity $\mathbf{u}_\mathrm{i}$ of the droplet interfaces $\partial\Omega_{\mathrm{d/a}}$ and $\partial\Omega_{\mathrm{d/le}}$ is related to the fluid velocity $\mathbf{u}$ by the kinematic boundary condition $(\mathbf{u}_\mathrm{i} - \mathbf{u}) \cdot \hat{\mathbf{n}} = 0$. Consequently, the normal velocity component $\mathbf{u}_\mathrm{mesh} \cdot \hat{\mathbf{n}}$ of the mesh vertices that follow the moving droplet/air interface $\partial\Omega_{\mathrm{d/a}}$, is given by $\mathbf{u} \cdot \hat{\mathbf{n}}$. By contrast, the normal mesh velocity component vanishes at the electrode $\partial\Omega_{\mathrm{d/le}}$ due to the kinematic boundary stated previously. Note that on the other hand the tangential velocity components of the mesh are not constrained by any physically motivated boundary conditions. In order to ensure that the mesh remains smooth at any computed time the resulting displacement of the mesh vertices representing the interfaces is expanded over the whole computational domain by means of a mesh smoothing method. In the present work the so-called Yeoh mesh smoothing is deployed. This method minimizes the mesh deformation energy assuming that the deforming domain behaves like a Yeoh hyperelastic material.

In the asymptotic limit $\mathbf{u} \to 0$ the Stokes equation yields the hydrostatic pressure distribution $p = p|_{z=0} - \Delta\rho g z$. In turn, the dynamic stress balance Eq. 6 converges to the (augmented) Young-Laplace equation

$$\Delta p = \tilde{\gamma} \, \kappa + \Delta\rho \, g \, z - \left([\![\mathbf{T}^\phi]\!] \cdot \hat{\mathbf{n}} \right) \cdot \hat{\mathbf{n}} \ . \tag{8}$$

we aim to solve. Here, the constant Laplace pressure $\Delta p = p|_{z=0} - p_\mathrm{a}$ can be interpreted as Lagrangian multiplier, which is determined by the constraint of constant volume or mass conservation. Furthermore, the NBC 5 at the footprint reduces to a trivial equality, while at the TCL the contact angle is fixed by the non-zero RHS of the GNBC 4. The parameters ρ^*, μ^*, and β^*, which determine the dynamic behavior of the equations above, have no physical meaning in the asymptotic limit and can be chosen arbitrarily in principle. However, in order to minimize inertial effects that slow down the time integration of the spatially discretized equations, a very high value was assigned to the dynamic viscosity, more specifically $\mu^* = 100\,\mathrm{Pa\,s}$, whereas the artificial density ρ^* was set to the actual mass density of the droplet ρ_d. As soon as the solution had converged towards the static equilibrium with respect to prescribed tolerance the time integration was terminated.

The velocity $\mathbf{u}$ and the pressure field p were spatially discretized using second-order Lagrangian elements. As long as it yields a converged solution the approach presented here works with any time-stepping method. The Newton method was utilized to solve the non-linear algebraic equations obtained by spatial discretization. Direct solvers were used to solve the resulting coupled system of linear equations.

Perfect dielectric vs. perfectly conducting droplets

In the main text, the droplets were supposed to be perfectly conducting bodies with isopotential surfaces. If we instead consider them as perfect dielectric bodies with finite permittivity and zero conductivity, the Young-Laplace equation appearing in the main text has to be replaced by the more general form Eq. 8 to account for dielectric media at both sides of the interface. Using the continuity conditions $[\![\mathbf{D}]\!] \cdot \hat{\mathbf{n}} = 0$ and $[\![\mathbf{E}]\!] \cdot \hat{\boldsymbol{\tau}}_i = 0$ at the droplet surface it can be shown that the electrostatic surface force density $\mathbf{f}^\phi$ is purely normal as it is also the case for perfectly conducting droplets (see above). Analogous to the limit of perfectly conducting droplets, the net repulsion force is obtained by integrating the electrostatic Maxwell stress in direction of the distance d over the droplet surface

$$F_\mathrm{r} = \hat{\mathbf{d}} \cdot \oint_{\partial\Omega_\mathrm{d}} \mathbf{T}^\phi \cdot \hat{\mathbf{n}}\,\mathrm{d}A = \hat{\mathbf{d}} \cdot \oint_{\partial\Omega_\mathrm{d}} \left[\varepsilon_0 \left(\mathbf{E} \cdot \hat{\mathbf{n}}\right) \mathbf{E} - \frac{1}{2} \varepsilon_0 \left(\mathbf{E} \cdot \mathbf{E}\right) \hat{\mathbf{n}}\right] \mathrm{d}A\,, \tag{9}$$

where $\mathbf{T}^\phi \cdot \hat{\mathbf{n}}$ is the Maxwell stress outside of the droplet. The relative permittivity of water is 78.4 [8]. As indicated in Fig. 4 for an exemplary parameter set, the computed repulsion forces between perfectly conducting and perfect dielectric droplets differ by less than 10 % at minimum d.

Strictly speaking, perfect dielectric media exhibit no free charges, which is definitively not the case for the water droplets used in experiments. In fact, in water charge generation occurs via auto-dissociation of molecules and beyond that, no special measures were taken to prevent the accumulation of free charges (ions) in a droplet. These free charges would contribute to the shielding of the external electric field within the droplets, such that $\mathbf{E} \to 0$. In summary, one can expect that any real droplets will range in an interval bounded by the ideal limits of perfectly conducting and perfect dielectric media.

Local distribution of electrostatic Maxwell stress for interacting droplets

As already mentioned in the main text, we assume that any non-axisymmetric influence that prevents the droplets from reaching static equilibrium can be neglected. With regard to the low contact angle hysteresis and vanishing inertial effects observed in the experiments, it seems justified to ignore the droplet motion in that context. However, so far it is questionable to what extent the mutual electrostatic interaction between the droplets induces deviations from axisymmetry. To highlight this issue, the electrostatic Maxwell stress at the interface of two rigid droplets is visualized in Fig. 5 for different values of d. It can be seen that in the limit $d \to \infty$ the stress distribution on the droplet surface converges to an axisymmetric distribution. By contrast, for decreasing d significant deviations from axisymmetry are found. However, the deviations are still relatively small compared to the Laplace pressure Δp and hence are not large enough to induce significant localized deformations. Therefore we expect the error in the surface deformation induced by ignoring the mutual droplet interaction to be negligible.

In Fig. 5 a visual comparison of perfectly spherical and equilibrium droplet shapes satisfying the Young-Laplace equation 8 is provided. We note that the opposing electrostatic Maxwell stress and gravitational acceleration causing the droplet to become prolate or oblate, respectively, balance such that the resulting shape can be hardly distinguished by eye from a perfectly spherical cap. For significantly smaller voltages or larger droplet volumes this will not be the case, such that the additional expense to solve the Young-Laplace equation seems justified.

Meshing and mesh convergence

As shown in Fig. 6, the vicinity of the droplets was tessellated with an unstructured mesh for reasons of flexibility. Structured meshes were used everywhere else. Systematic mesh convergence studies were performed to demonstrate the mesh independence of the solutions presented in this work. An estimate of the mesh-independent solution can be obtained from the solutions based on three uniformly, recursively refined meshes by extrapolation. In the present context *recursively* means that all nodes of the initial mesh form a subset of the nodes of the refined mesh. Let ψ_1, ψ_2, and ψ_3 denote the numerical solutions for a local or global quantity ψ based on recursively refined meshes with local element sizes h_1, h_2, and h_3, respectively. To be more precise, the length of the longest edge of each element was used as a measure for the locally varying element size. In the asymptotic range the mesh-dependent solutions ψ_i can be expanded around the exact solution ψ as

$$\psi_i = \psi + C h_i^p + \mathcal{O}\left(h_i^{p+1}\right) , \quad i = 1,\, 2,\, 3 . \tag{10}$$

where p is the theoretical order of accuracy of the numerical scheme, which is unknown to us. Ignoring any higher-order terms in the equation above the exact (mesh-independent) solution can be estimated by

$$\psi^* = \psi_3 + \frac{\psi_3 - \psi_2}{r^{p^*} - 1} , \tag{11}$$

with the observed order of accuracy

$$p^* = \ln\left(\frac{\psi_1 - \psi_2}{\psi_2 - \psi_3}\right) / \ln(r) . \tag{12}$$

Here, the asterisk highlights estimated quantities and $r = h_2/h_3 = h_1/h_2 = 2$ denotes the mesh-refinement factor. We note that p^* and ψ^* are independent of the absolute, local element size h_i. They rather depend on the mesh-refinement factor r, which is uniform throughout the whole computational domain regardless of whether the mesh is isotropic or anisotropic as long as the refinement itself is uniform.

As representative global quantities ψ for mesh convergence we chose the equilibrium Laplace pressure Δp in Eq. 8 and the repulsion force F_r for the axisymmetric and three-dimensional computations, respectively. In Fig. 7 the relative error $|\psi_i - \psi^*| / |\psi^*|$ in each of these quantities is shown as a function of the uniform, global mesh refinement level h_i/h_ref, where we have identified the coarsest mesh as reference, i.e. $h_\mathrm{ref} \equiv h_1$, and thus $h_i/h_\mathrm{ref} = \{1,\, 0.5,\, 0.25\}$ for $i = \{1,\, 2,\, 3\}$. Evidently, the solutions converge asymptotically towards mesh-independent configurations. Furthermore, the results reveal that the spatial resolution of the reference meshes, which are depicted in Fig. 6 for exemplary values of the droplet volume and the distance d, is sufficient, as the relative error in each of the quantities is less than 1 %.

Computation of the repulsion force based on the virtual work principle

Instead of integrating the electrostatic Maxwell stress over the droplet surface, one can exploit the virtual work principle to compute the repulsion force. According to this principle, the unknown repulsion force is given by the change of the electrostatic potential energy δW stored in the electric field with respect to virtual displacements δd:

$$F_\mathrm{r} = \left.\frac{\delta W}{\delta d}\right|_\phi . \tag{13}$$

Here, the subscript ϕ indicates that the potential at the boundaries is kept fixed during displacement. Assuming conducting droplets the electrostatic potential energy reads

$$W = \frac{1}{2}\varepsilon_0 \int_{\Omega_\mathrm{a}} (\mathbf{E} \cdot \mathbf{E})\, \mathrm{d}V , \tag{14}$$

where Ω_a denotes the domain occupied by air with permittivity ε_0. Within COMSOL Multiphysics® [9] the derivative in Eq. 13 can be easily computed by means of a built-in sensitivity analysis for certain values of d. However, this requires at least one of the droplet interfaces to be tessellated with a moving mesh such that it can be translated in the lateral direction with respect to the control variable d. This might increase the total number of degrees of freedom N_dof significantly, depending on the number of movable mesh elements. In the present work, Laplacian mesh smoothing was applied on quadratic Lagrangian elements. Moreover, the calculation of the derivative in Eq. 13 itself requires at least one additional linear system of equations with dimension $N_\mathrm{dof} \times N_\mathrm{dof}$ to be solved which increases computational costs further. Apart from these drawbacks concerning computational efficiency in comparison with the integration of the local electrostatic Maxwell stress, both approaches should yield the same repulsion force in theory. In fact, for the problem under study the difference is negligible with respect to the desired accuracy.

Model of interacting dipoles

In the following, a semi-analytical model that provides easy access to the repulsion force with decent accuracy will be derived. This model relies on the assumption that the repulsion force between two sessile droplets in a parallel-plate capacitor can be deduced from the force between two interacting, induced dipoles. For clarification, we refer to Fig. 8 where the model is motivated using simple schematics. Evidently, based on symmetry arguments the setups shown in Fig. 8a and 8b are perfectly equivalent with regard to the electrostatic potential. The electrostatic potential around the mirror-fused bodies Ω_1 and Ω_2 is antisymmetric with respect to the z-coordinate, i.e. $\phi(x, y, z) = -\phi(x, y, -z)$. Thus, the lower electrode in the original problem, where we impose the Dirichlet condition $\phi = 0$, can be interpreted as a symmetry plane. We note that the force acting on each of the bodies is twice the force acting on the bodies in the original problem.

In order to proceed we substitute the electrodes at $z = -h$ and $z = h$ by a uniform electric field at infinity $\lim_{r\to\infty} \mathbf{E} = E_0\hat{\mathbf{z}}$, where the electric field strength E_0 is given by $-\Delta\phi/h$. This simplification is reasonable as long as the characteristic dimension of the droplets is much smaller than the electrode spacing h, such that the perturbation of the applied uniform electric field owing to the presence of the droplets is negligibly small at $z = \pm h$ where the electrodes are originally located. The essence of the model is to represent the mirror-fused bodies by point dipoles, as shown in Fig. 8c. The electric field each point dipole is exposed to is the superposition of the applied field and dipole field due to the neighboring body. Since the dipoles are induced, their strength depends on the local electric field value. The dipole moment induced by an external electric field of strength E_0 pointing in z-direction is given by

$$\mathbf{p}_i = \hat{\mathbf{z}}\, 4\pi\varepsilon\alpha_i E_0 \,, \quad i = 1,\, 2 \,. \tag{15}$$

Here, $\varepsilon = \varepsilon_0\varepsilon_\mathrm{r}$ denotes the dielectric constant of the medium the droplets/dipoles are embedded in. To account for interacting dipoles, the electric field must be expanded in an infinite series of recursively calculated contributions of the dipoles. These iterative calculations have been already carried out by Stoy [10], who provides the following closed-form expression for the dipole moments:

$$\mathbf{p}_1 = \hat{\mathbf{z}}\, 4\pi\varepsilon\alpha_1 E_0 \left(\frac{1-\alpha_2/d^3}{1-\alpha_1\alpha_2/d^6}\right) \,, \quad \text{and} \quad \mathbf{p}_2 = \hat{\mathbf{z}}\, 4\pi\varepsilon\alpha_2 E_0 \left(\frac{1-\alpha_1/d^3}{1-\alpha_1\alpha_2/d^6}\right) \,. \tag{16}$$

For $d \to \infty$ the factor in brackets that accounts for the reciprocal electrostatic induction approaches unity, and the induced dipole moments converge to those of isolated dipoles given by Eq. 15. So far the coefficients α_1 and α_2, solely depending on the geometry of the mirror-fused bodies, are undetermined. To proceed we assume that the conducting droplets are perfectly spherical and hence the mirror-fused bodies can be obtained from merging two spherical caps. According to Felderhof & Palaniappan [11], the geometric coefficient α_i for such a body is given by

$$\alpha_i = 8\sin^3\theta_i a_i^3 \int_0^\infty \tau^2 \left(\frac{\tanh \pi\tau}{\tanh(\pi-\theta_i)\,\tau} - 1\right) \mathrm{d}\tau \,, \quad i = 1,\, 2 \,. \tag{17}$$

The radius a_i and the volume of the spherical droplet V_i are related by

$$a_i^3 = \frac{3V_i}{\pi}\frac{1}{2-3\cos\theta_i+\cos^3\theta_i} \,, \quad i = 1,\, 2 \,. \tag{18}$$

Accordingly, Eq. 17 can be rewritten as

$$\alpha_i = \frac{24V_i}{\pi}\frac{\sin^3\theta_i}{2-3\cos\theta_i+\cos^3\theta_i} \int_0^\infty \tau^2 \left(\frac{\tanh \pi\tau}{\tanh(\pi-\theta_i)\,\tau} - 1\right) \mathrm{d}\tau \,, \quad i = 1,\, 2 \,. \tag{19}$$

The improper integral depends on the contact angle only and can be evaluated numerically. It should not go unmentioned that the relation for α given above is only valid for droplets with contact angles between 90° and 180°. In the case $\theta = 90°$, the bodies become simple spheres, and the geometric coefficient simplifies to a^3. Rearranging of Eqs. 16 and 19 yields the expressions presented in the main text. The equation given above implies that the point dipoles are located at the centers of the circles of intersection, i.e. at the intersection between the plane $z = 0$ and the axis of rotation of the mirror-fused bodies.

Before we address the calculation of the repulsion force we elaborate on the electrostatic potential derived from the present model. The potential corresponding to the uniform, undisturbed applied electric field is

$$\phi_0 = -E_0 z \,. \tag{20}$$

The potentials due to the dipoles, in turn, are given by

$$\phi_i = -\mathbf{p}_i \cdot \boldsymbol{\nabla}_i \left(\frac{1}{4\pi\varepsilon r_i} \right) = \frac{\mathbf{p}_i \cdot \mathbf{r}_i}{4\pi\varepsilon r_i^3} \,, \qquad i = 1,\, 2 \tag{21}$$

with

$$\mathbf{r}_1 = \left(x + \frac{d}{2} \right) \hat{\mathbf{x}} + y\hat{\mathbf{y}} + z\hat{\mathbf{z}} \,, \quad \text{and} \quad \mathbf{r}_2 = \left(x - \frac{d}{2} \right) \hat{\mathbf{x}} + y\hat{\mathbf{y}} + z\hat{\mathbf{z}} \,, \tag{22}$$

and

$$r_1 \equiv |\mathbf{r}_1| = \sqrt{\left(x + \frac{d}{2} \right)^2 + y^2 + z^2} \,, \quad \text{and} \quad r_2 \equiv |\mathbf{r}_2| = \sqrt{\left(x - \frac{d}{2} \right)^2 + y^2 + z^2} \,. \tag{23}$$

In Fig. 8c isolines of the combined potential

$$\phi^* = \begin{cases} \phi_0 + \phi_2 = -E_0 z + \dfrac{\mathbf{p}_2 \cdot \mathbf{r}_2}{4\pi\varepsilon r_2^3} \,, & \forall\, \mathbf{x} \in \Omega_1 \\ \phi_0 + \phi_1 = -E_0 z + \dfrac{\mathbf{p}_1 \cdot \mathbf{r}_1}{4\pi\varepsilon r_1^3} \,, & \forall\, \mathbf{x} \in \Omega_2 \\ \phi_0 + \phi_1 + \phi_2 = -E_0 z + \dfrac{\mathbf{p}_1 \cdot \mathbf{r}_1}{4\pi\varepsilon r_1^3} + \dfrac{\mathbf{p}_2 \cdot \mathbf{r}_2}{4\pi\varepsilon r_2^3} \,, & \forall\, \mathbf{x} \in \mathbb{R}^3 \setminus (\Omega_1 \cup \Omega_2) \end{cases} \tag{24}$$

in the xz-plane are plotted. In the expression above, Ω_1 and Ω_2 denote the domains of the mirror-fused bodies. This piecewise-defined potential takes into account that the dipoles cannot induce a field on themselves, and hence the electric field each dipole experiences is solely given by the uniform applied electric field and the field of the neighboring dipole. One may notice that the latter contribution gives rise to slightly bent isolines around each dipole. Furthermore, owing to the neglected higher multipole moments the approximate potential ϕ^* does not become zero at the surfaces of the mirror-fused bodies, as implied by the exact boundary condition $\phi = 0$. Actually, if and only if the contact angle θ is 90° and the droplets are infinitely far away from each other, i.e. $d \to \infty$, the potential ϕ^* vanishes at the surface of the droplets.

In general, the force exerted on a point dipole is given by

$$\mathbf{F} = (\mathbf{p} \cdot \boldsymbol{\nabla})\, \mathbf{E} \,. \tag{25}$$

In the present case this relation simplifies to

$$\mathbf{F}_{\mathrm{r}}{}^* = \hat{\mathbf{d}} \frac{3\, \mathbf{p}_1 \cdot \mathbf{p}_2}{4\pi\varepsilon d^4} \,, \tag{26}$$

where $\hat{\mathbf{d}}$ is the normalized distance vector defined by $\hat{\mathbf{d}} \equiv \mathbf{d}/\,|\mathbf{d}| = \mathbf{d}/d$. Recalling that the force between the droplets is just one half of the force between the mirror-fused bodies, we obtain

$$\mathbf{F}_{\mathrm{r}} = \hat{\mathbf{d}} \frac{3\, \mathbf{p}_1 \cdot \mathbf{p}_2}{8\pi\varepsilon d^4} \,. \tag{27}$$

Inserting Eqs. 16 and 19 in the relation above eventually yields the desired repulsion force.
The present model of interacting dipoles is a first-order model since any higher multipole moments are neglected. Higher multipole moments have a twofold origin. On the one hand, they result from the finite extension of the polarizable body. On the other hand, they depend on the shape of a body, meaning that only for an isolated spherical shape all higher multipole moments vanish. Therefore, the present model is expected to give accurate results only if d is much larger than the radii of the spherical droplets a_1, and a_2 and the contact angle does not deviate much from 90°.

[1] Daniel, D., Timonen, J. V. I., Li, R., Velling, S. J. & Aizenberg, J. Oleoplaning droplets on lubricated surfaces. *Nature Physics* **13**, 1020–1025 (2017).

[2] Kajiya, T. *et al.* Cylindrical chains of water drops condensing on microstructured lubricant-infused surfaces. *Soft Matter* **12**, 9377–9382 (2016).
[3] Kreder, M. J. *et al.* Film dynamics and lubricant depletion by droplets moving on lubricated surfaces. *Physical Review X* **8**, 031053 (2018).
[4] Emslie, A. G., Bonner, F. T. & Peck, L. G. Flow of a viscous liquid on a rotating disk. *Journal of Applied Physics* **29**, 858–862 (1958).
[5] The MathWorks® Inc. MATLAB®, Release 2017b (2017). Natick, MA, United States.
[6] Sinn, N., Schür, M. T. & Hardt, S. No-contact electrostatic manipulation of droplets on liquid-infused surfaces: experiments and numerical simulations. *Applied Physics Letters* **114**, 213704 (2019).
[7] Gerbeau, J.-F. & Lelièvre, T. Generalized Navier boundary condition and geometric conservation law for surface tension. *Computer Methods in Applied Mechanics and Engineering* **198**, 644–656 (2009).
[8] Fernández, D. P., Mulev, Y., Goodwin, A. R. H. & Sengers, J. M. H. L. A database for the static dielectric constant of water and steam. *Journal of Physical and Chemical Reference Data* **24**, 33–70 (1995).
[9] COMSOL AB. COMSOL Multiphysics®, Version 5.5 (2019). Stockholm, Sweden.
[10] Stoy, R. D. Interactive dipole model for two-sphere system. *Journal of Electrostatics* **33**, 385–392 (1994).
[11] Felderhof, B. U. & Palaniappan, D. Longitudinal and transverse polarizability of the conducting double sphere. *Journal of Applied Physics* **88**, 4947–4952 (2000).
