# Supplementary material for: Manipulation and control of droplets on surfaces in a homogeneous electric field
Source: Nat Commun. 2022 Jan 12;13:289. doi: 10.1038/s41467-021-27879-0 (PMC8755840; doi:10.1038/s41467-021-27879-0)
Supplement: Supplementary file 3 — Description of Additional Supplementary Files [file 41467_2021_27879_MOESM3_ESM.pdf]

Description of Additional Supplementary Files

File Name: Supplementary Movie 1
Description: Experiment with 10 µL immobilized droplet and two 6.25 µL moving droplets. The electric field is turned off. Both moving droplets slide down the inclined surface along the y-direction. As the center-to-center distance of the second moving and the immobilized droplet in x-direction is smaller than the sum of each of their radii, they eventually coalesce as they approach each other.

File Name: Supplementary Movie 2
Description: Experiment with 10 µL immobilized droplet and a 10 µL moving droplet. The applied potential is 4.5 kV. First, the moving droplet slides down the inclined surface along the y-direction. At the beginning, the center-to-center distance of the moving and the immobilized droplet in x-direction is smaller than the sum of each of their radii. As the moving droplet approaches the immobilized one it is accelerated in the positive x-direction and is decelerated in y-direction. Eventually, the moving droplet passes the immobilized one without coalescing because of the mutual electrostatic repulsion force.

File Name: Supplementary Movie 3
Description: Experiment with a 10 µL immobilized droplet and a 35 µL moving droplet. The applied potential is 5.5 kV. The video was slowed down 16 times compared to its original speed. The white line was added to the video to mark the initial x-position of the moving droplet's edge. When the large mobile droplet approaches the immobilized one, a shift of the mobile droplet in a negative x-direction can be seen. The two droplets would probably not have coalesced without an electric field. Still, this video shows that the larger mobile droplet is also effectively repelled by the smaller immobile one.

File Name: Supplementary Movie 4
Description: Experiment with a total of 48 20 µL droplets applied with a pipette on a LIS with zero inclination. No electric field is applied. A lateral airflow is guided through a hole in the ITO-wafer mount on the left. By the airflow droplets are pushed to the right side of the LIS where they coalesce, as expected, with other droplets that are not displaced by the airflow.

File Name: Supplementary Movie 5
Description: Experiment with a total of 48 20 µL droplets applied with a pipette on a LIS with zero inclination. The applied potential is 5.5 kV with a capacitor plate distance of 3.5 mm. A lateral airflow (same conditions as in movie 4) is guided through a hole in the ITO-wafer mount on the left. No coalescence can be observed.

File Name: Supplementary Movie 6
Description: Experiment showing the sampling from a droplet array. Using a pipette 14 droplets with volume of 30 µL are dispensed LIS with zero inclination. The side of the fused-silica wafer facing the LIS was powdered with rhodamine B prior to the experiment. The dye is visible in faint green. At 1 s a potential of 4 kV is applied between the electrodes of the plate capacitor with an air gap width of 7 mm. Starting at 7 s the applied voltage is ramped up continuously to 6 kV within 3 s. Tiny secondary droplets detach from the apex of the

sessile droplets on the LIS at 9 s and are accelerated towards the counter electrode. Eventually, these secondary droplets become visible as small magenta patches.
